# Supplementary material for: High-spin Co3+ in cobalt oxyhydroxide for efficient water oxidation
Source: Nat Commun. 2024 Feb 15;15:1383. doi: 10.1038/s41467-024-45702-4 (PMC10869355; doi:10.1038/s41467-024-45702-4)
Supplement: Supplementary file 1 — Supplementary Information [file 41467_2024_45702_MOESM1_ESM.pdf]

Supplemental Information

High-Spin Co^{3+} in Cobalt Oxyhydroxide for Efficient Water Oxidation

Xin Zhang[1#], Haoyin Zhong[1#], Qi Zhang[1], Qihan Zhang[1], Chao Wu[23], Junchen Yu[1], Yifan Ma[1], Hang An[1], Hao Wang[1], Yiming Zou[4], Caozheng Diao[5], Jingsheng Chen[1], Zhi Gen Yu[6], Shibo Xi[2*], Xiaopeng Wang[1378*], Junmin Xue[1*]

Affiliations

[1]Department of Materials Science and Engineering, National University of Singapore, Singapore, 117575.

[2]Institute of Sustainability for Chemical, Energy and Environment (ISCE), Agency for Science, Technology and Research (A*STAR), Singapore, 627833.

[3]College of Materials Science and Engineering, Sichuan University, Chengdu, China, 610065.

[4]School of Materials Science and Engineering, Nanyang Technological University, Singapore, 639798.

[5]Singapore Synchrotron Light Sources (SSLS), National University of Singapore, Singapore, 117603.

[6]Institute of High Performance Computing (IHPC), Agency for Science, Technology and Research (A*STAR), Singapore, 138632.

[7]State Key Laboratory of Intelligent Construction and Healthy Operation and Maintenance of Deep Underground Engineering, Sichuan University, Chengdu, China, 610065.

[8]Tefusen Semiconductor & Hydrogen Energy Technology (Yunnan) Co., Ltd, Wenshan Zhuang and Miao Autonomous Prefecture, China, 663200.

#Equal contribution

*Corresponding to msexuejm@nus.edu.sg (Junmin Xue); msewxia@nus.edu.sg (Xiaopeng Wang); Xi_shibo@isce2.a-star.edu.sg (Shibo Xi)

Supplementary Figures S1-27

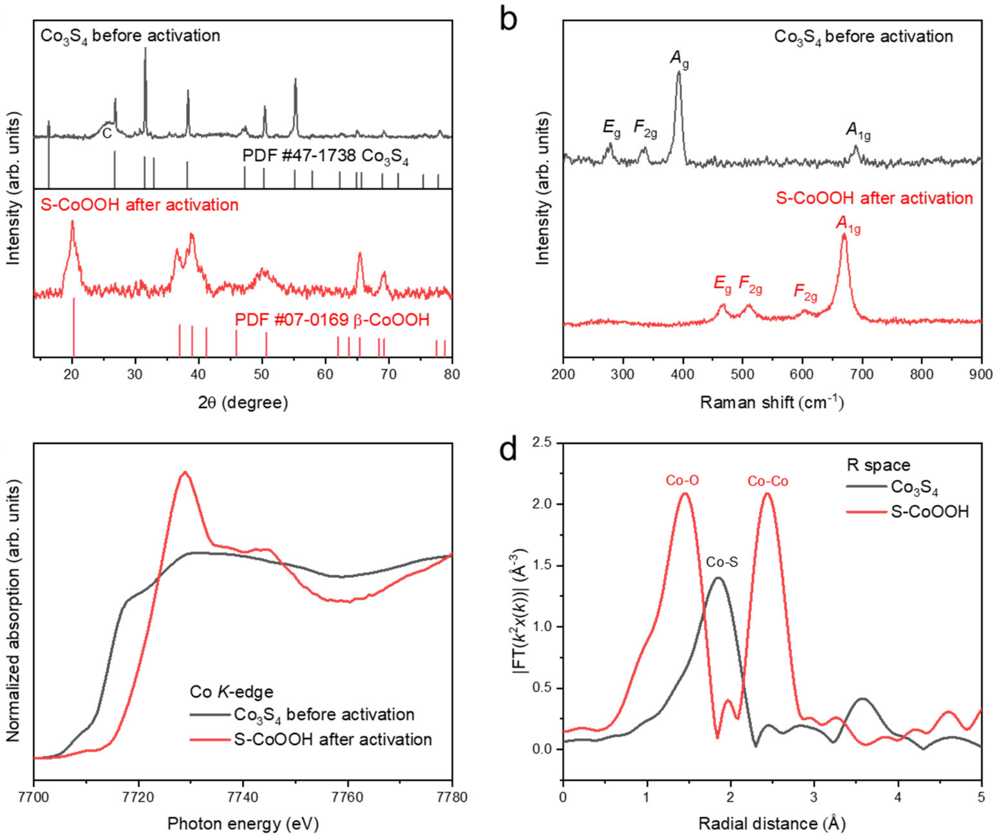

Supplementary Fig. S1 Structural characterization of as-prepared Co_3S_4 and after activation S-CoOOH. a XRD patterns of Co_3S_4 and S-CoOOH with standard peaks of Co_3S_4 and β-CoOOH listed. **b** Raman spectra of Co_3S_4 and S-CoOOH. **c** Normalized Co *K*-edge XAS spectra of Co_3S_4 and S-CoOOH. **d** FT-EXAFS spectra of Co *K*-edge of Co_3S_4 and S-CoOOH.

The CoOOH sample was synthesized through electro-oxidation of cobalt sulfides (more details are given in the Methods section). For the comparison, the counterpart chooses β-$Co(OH)_2$ as the precursor. The cobalt sulfides and after electro-oxidation samples are analyzed via X-ray diffraction (XRD), Raman spectroscopy, and X-ray absorption spectroscopy (XAS) (Supplementary Fig. S1). The XRD results show that the cobalt sulfide sample is indexed to cubic Co_3S_4 (JCPDS 47-1738) and after electro-oxidation, the pre-catalyst Co_3S_4 is fully re-constructed into rhombohedral β-CoOOH (R-3m space group, JCPDS 07-0169) (Supplementary Fig. S1a).

This is further confirmed by Raman spectra (Supplementary Fig. S1b), showing the peaks

of 282 cm^{-1} and 392 cm^{-1} in the cobalt sulfide sample, which are attributed to the asymmetric bending vibration of tetra S-Co bond and S-S pair, respectively[1,2]. After electro-oxidation, these two peaks completely disappear and the peaks at 502 cm^{-1} and 684 cm^{-1} are detected, corresponding to E_g and A_{1g} vibration modes of Co-O in CoOOH[3]. The electro-oxidation sample is named as S-CoOOH. Such a spectrum is commonly attributed to β-CoOOH.

In addition, the variation in crystal structure of Co_3S_4 bulk before and after the OER process is studied using XAS at Co *K*-edge. Shown in Supplementary Fig. S1c, the reconstruction-derived S-CoOOH sample, compared to the pre-catalyst Co_3S_4, exhibits obvious redshifts in the absorption thresholds and much higher white line intensity (at 7728 eV). It indicates that the electron state around the Fermi level changes from a metallic state to a localized state, revealing a significant phase reconstruction[4]. It is further verified by Co *K*-edge Fourier transformed extended X-ray near fine structure (FT-EXAFS) spectra (Supplementary Fig. S1d). The peaks belonging to Co-S bonds in the pre-catalyst Co_3S_4 completely disappear after the long-term chronopotentiometry treatment, and simultaneously Co-O and Co-Co bonds are observed in the reconstruction-derived S-CoOOH compounds.

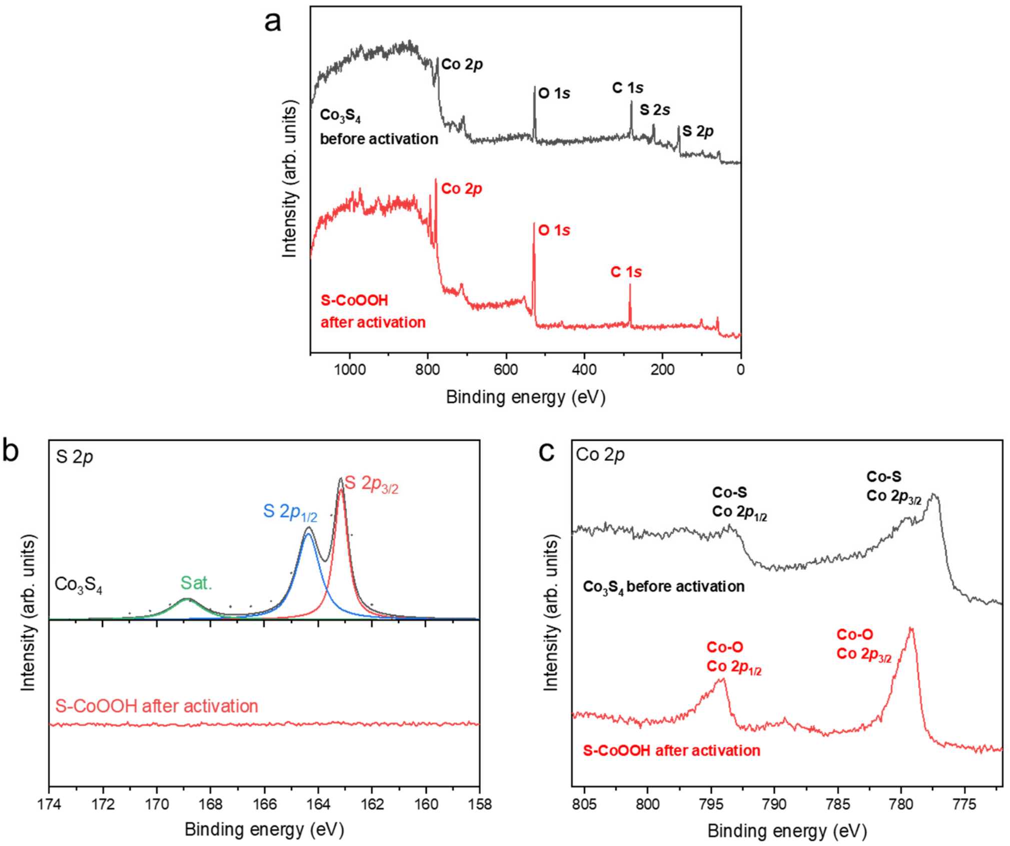

Supplementary Fig. S2 XPS spectra of Co_3S_4 before and after the OER measurement: a survey in a wide range, and high-resolution spectra of S 2*p* (**b**), Co 2*p* (**c**).

X-ray photoelectron spectroscopy (XPS) is carried out to verify no S signals on the S-CoOOH surface (in-depth 5-10 nm). Supplementary Fig. S2a is the wide-range spectra of as-precursor Co_3S_4 and after-activation S-CoOOH. The peaks at binding energies of 160, 221, 279, 527, and 780 eV are assigned to S 2*p*, S 2*s*, C 1*s*, O 1*s*, and Co 2*p*[5], respectively. However, these two signals belonging to S 2*p* and S 2*s* disappear completely in S-CoOOH after a long time OER process.

Besides, Supplementary Fig. S2b is the high-resolution XPS spectra of S 2*p* for Co_3S_4 before and after the OER measurement. The XPS S 2*p* spectrum in as-precursor Co_3S_4 before activation can be deconvoluted into two main peaks along with a shakeup satellite, in which the peaks located at 163.1 eV for S $2p_{3/2}$ and 164.4 eV for S $2p_{1/2}$, agree with the Co-S bonds[3]. The existence of the satellite may be related to the partial oxidation of sulfur species in the air on the sample surface. After 10 hours of chronopotentiometry treatment, no peaks are assigned to

S 2*p* in S-CoOOH, indicating that sulfide is totally removed during the OER process.

In addition, the high-resolution spectra of Co 2*p* for Co_3S_4 before and after the OER measurement are shown in Supplementary Fig. S2c. These two peaks with the binding energy of 794.3 eV and 779.1 eV belong to Co $2p_{1/2}$ and $2p_{3/2}$[5], where the signals are attributed to Co-S bonds in Co_3S_4 precursor. Notably, after the OER process, the peaks of Co 2*p* in S-CoOOH shift to higher binding energies compared to those in Co_3S_4. This shift is indicative of a transition from Co-S to Co-O bonding and also means a higher valance state of Co in S-CoOOH.

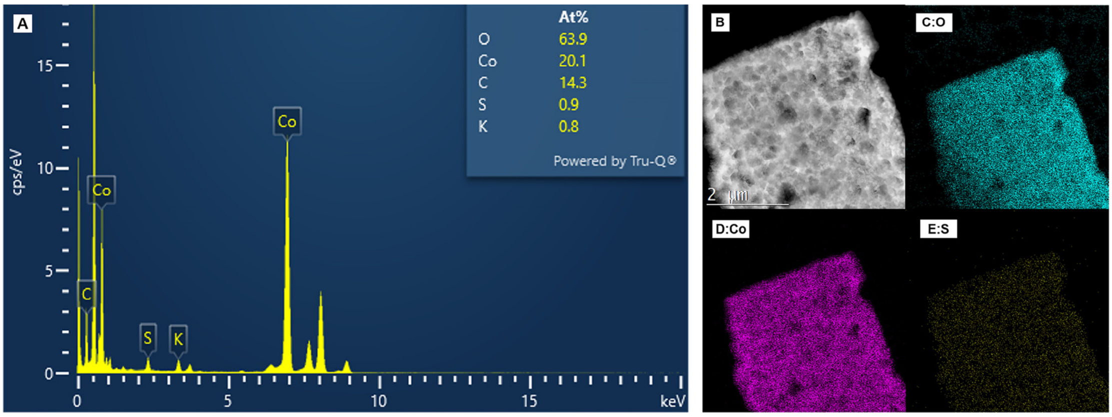

Supplementary Fig. S3 The STEM-EDS results of S-CoOOH. a The quantitative elemental analysis. **b** EDS analysis area. **c-e** EDS mapping of oxygen (dark cyan), cobalt (purple), sulfur (yellow). No S signal is detected. "cps" is an abbreviation for "Counts Per Second". "At%" stands for "atomic percent".

In the next section, the compositions of the precursor cobalt sulfide and reconstruction-derived S-CoOOH are analyzed using energy dispersive X-ray spectroscopy (EDS) in both scanning transmission electron microscopy (in a high-angle annular dark field model, STEM-HAADF) and scanning electron microscopy (SEM), high-resolution transmission electron microscopy (HR-TEM), and inductively coupled plasma (ICP) measurements.

Shown in Supplementary Fig. S3, STEM-EDS mappings of reconstruction-derived S-CoOOH, Co and O are uniformly distributed corresponding with the rectangle-shaped sample. Interestingly, nearly negligible signal peaks attributed to S are detected, given by the quantitative EDS results based on both STEM and SEM images (Supplementary Fig. S3, S4). Also, ICP measurements are served as supplemental evidence (Supplementary Table S1). In contrast, SEM and the corresponding EDS mapping of precursor cobalt sulfide show the uniform distribution of excess S element in the whole range (Supplementary Fig. S5).

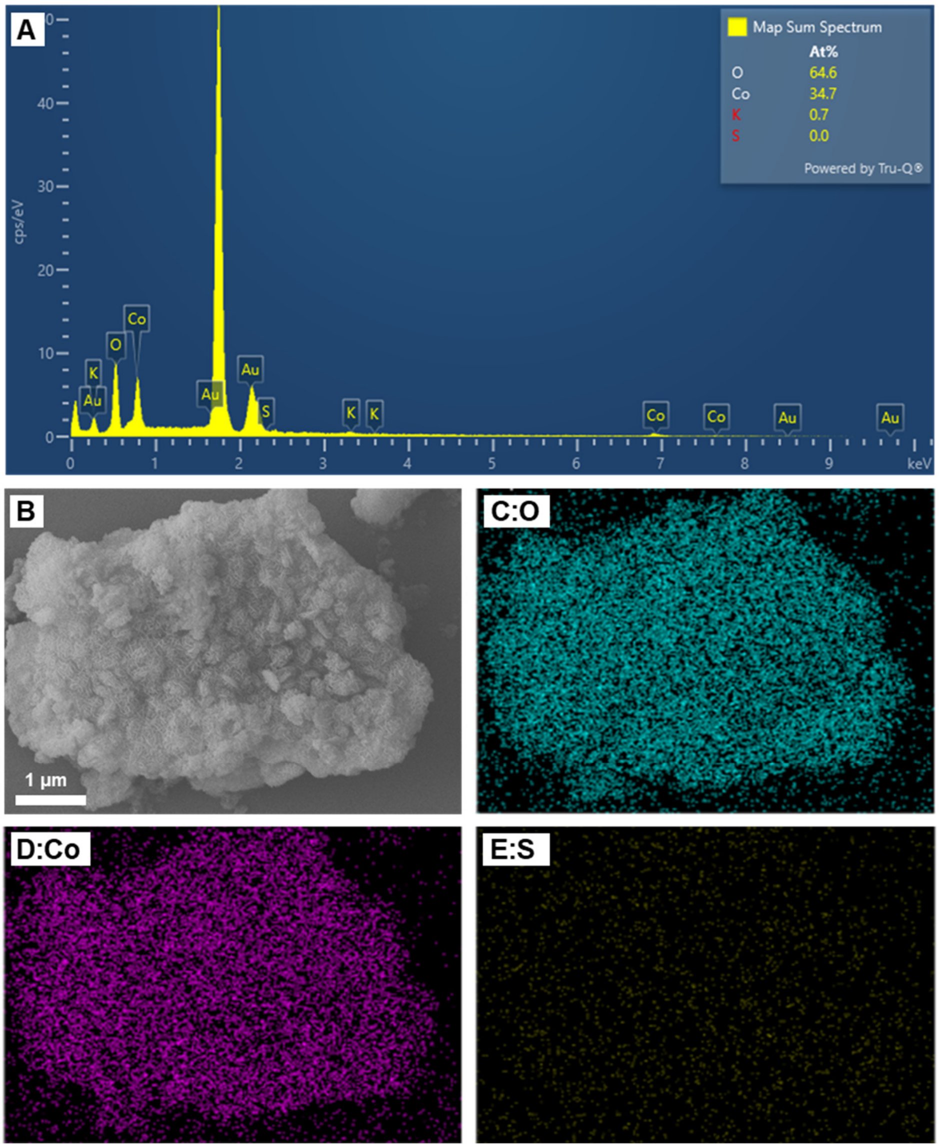

Supplementary Fig. S4 The SEM-EDS results of S-CoOOH. a The quantitative elemental analysis. **b** EDS analysis area. **c-e** EDS mapping of oxygen (dark cyan), cobalt (purple), sulfur (yellow). No S signal is detected. “cps” is an abbreviation for “Counts Per Second”. “At%” stands for “atomic percent”.

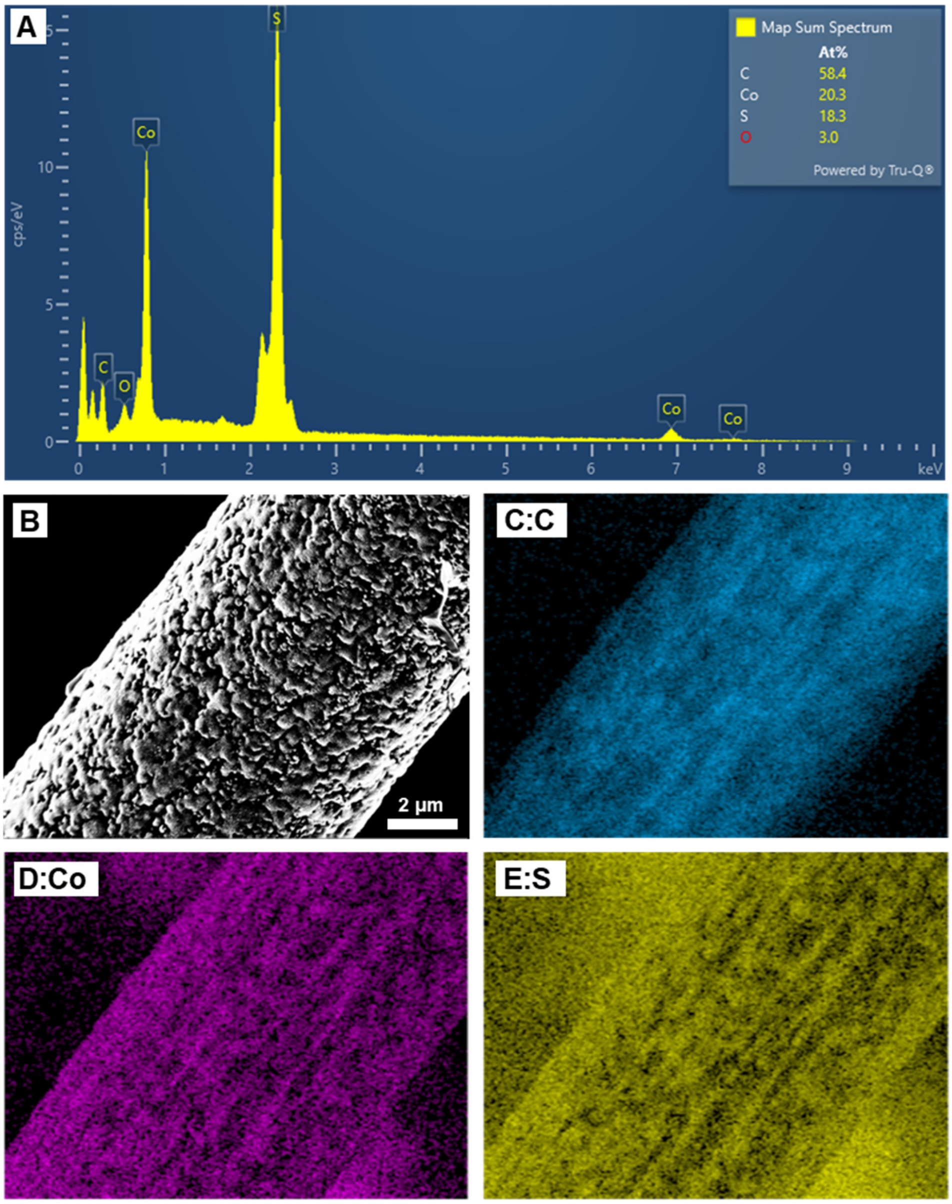

Supplementary Fig. S5 The SEM-EDS results of the precursor cobalt sulfide. a The quantitative elemental analysis. **b** EDS analysis area. **c-e** EDS mapping of carbon (cyan blue), cobalt (purple), sulfur (yellow). "cps" is an abbreviation for "Counts Per Second". "At%" stands for "atomic percent".

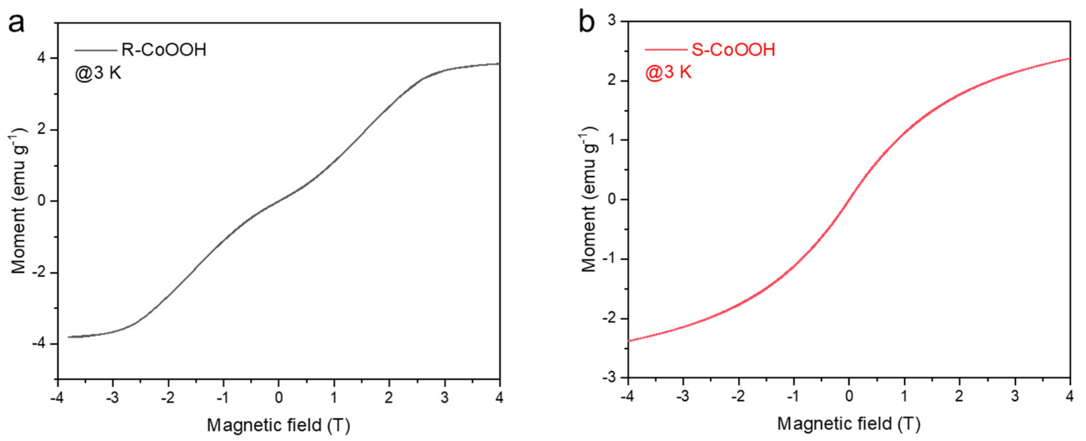

Supplementary Fig. S6 Magnetic hysteresis (**M-H**) loop at 3 K of R-CoOOH (**a**) and S-CoOOH (**b**).

Supplementary Fig. S6 is the magnetic hysteresis (**M-H**) loops of R-CoOOH and S-CoOOH recorded at 3 K. The nonlinear **M-H** loop at 3 K for R-CoOOH might be due to the following reasons: **1. The decreased antiferromagnetic coupling effect.** The **M-H** loop measurement was conducted at 3 K, which was close to the Néel temperature (around 10 K, Fig. 2d). Near the Néel temperature, the antiferromagnetic coupling would weaken, leading to a non-linear shift. Such a phenomenon of anomalous non-linear **M-H** loops near the Néel temperature has been reported in many antiferromagnetic materials, *i.e.* $EuNiGe_3$[6], $C_{13}H_{13}Ba_4ClCo_3O_{26}$[7]. **2. A spin-flop transition.** The spin-flop transition generally occurs in antiferromagnetic materials that have weak magnetic anisotropy. Lee et al. proposed that the non-linear deviation observed in the antiferromagnetic material $Co_4Ta_2O_9$ was attributed to a spin-flop transition at **Hc** $\approx$ 0.3 T for an applied field[8].

For S-CoOOH, why both the remanent magnetization and the coercive field are reduced to zero might be due to the following possible reasons. There is only a quite small number of high-spin state Co^{3+} atoms, which contributes fixed magnetic moments and ferromagnetic signals in S-CoOOH. At low temperatures, the paramagnetic contribution from the low-spin state Co^{3+} might dominate the whole magnetic phenomenon, which would finally result in the negligible remanent magnetization and coercive field observed at 3 K. This would be further explored in our further work.

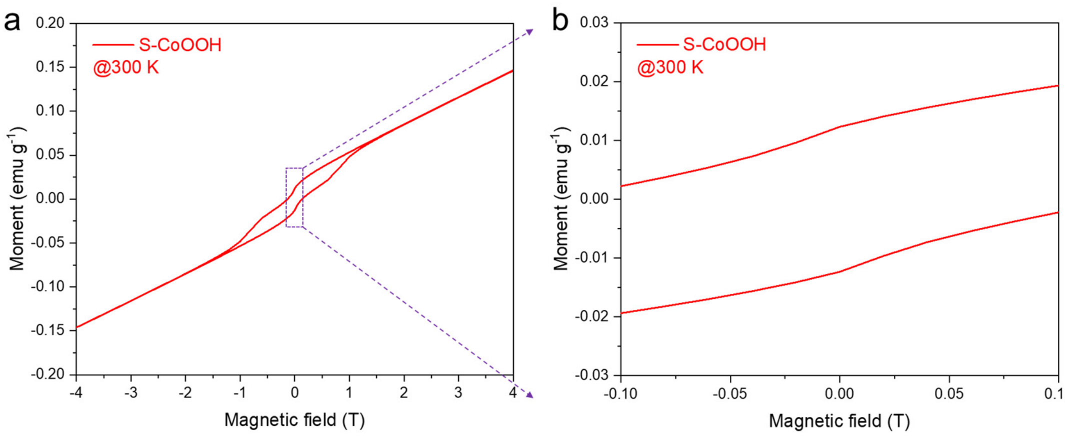

Supplementary Fig. S7 a Magnetic hysteresis (**M-H**) loop of S-CoOOH, recorded at 300 K. **b** The enlarged result of Supplementary Fig. S7a, between -0.10 and 0.10 T (-1000 Oe and 1000 Oe).

The slight divergences between ZFC (zero field cooled) and FC (field cooled) curves of R-CoOOH and S-CoOOH could be explained as below: For R-CoOOH, it exhibits paramagnetic behavior above the Néel temperature and antiferromagnetic behavior below the Néel temperature. Due to the applied magnetic field of 100 Oe for the FC curve, the relatively weak moment is induced, hence the slight divergence between the ZFC and FC curves is observed above the Néel temperature (Fig. 2c). In addition, a significant divergence between the ZFC and FC curves near the Néel temperature (about 10 K) is attributed to the transition between paramagnetic and antiferromagnetic properties.

For S-CoOOH, the slight divergence between the ZFC and FC curves in the high-temperature region (~100-300 K) may be due to the small coercive field of S-CoOOH. This hypothesis could be verified by the **M-H** loop of S-CoOOH at 300 K (Supplementary Fig. S7a). The moment at 0.01 T (100 Oe, indicative of FC at 300 K) is slightly higher than at 0 T (indicative of ZFC at 300 K) (Supplementary Fig. S7b). An applied magnetic field of 100 Oe, which is near the coercive field and will tilt moment along the magnetic field direction, might lead to a slight divergence between the ZFC and FC curves. Moreover, the increased trend noted in the FC curve at low temperatures (~3-50 K) can be attributed to the paramagnetic contribution from the low-spin state Co^{3+}, similar to behaviors observed in the previously reported partially high-spin state $LaCoO_3$[9].

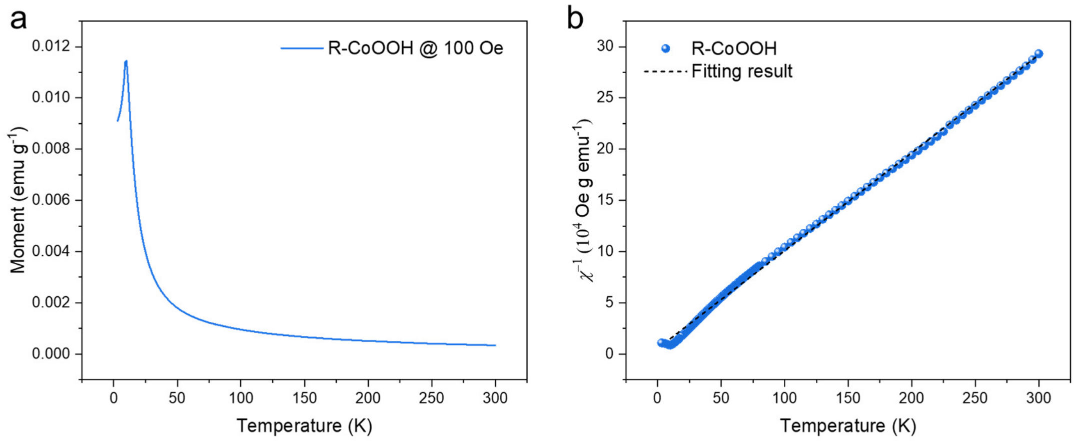

Supplementary Fig. S8 Magnetic property of R-CoOOH. a Temperature-dependent magnetization (**M**-T) under **H** = 100 Oe. **b** The temperature dependence inverse susceptibility (χ^{-1}). The dotted line is the fitting result by a Curie-Weiss law.

The temperature-dependent magnetizations (**M**-T) were measured with a magnetic field of **H** = 100 Oe under field-cooling procedures for R-CoOOH (Supplementary Fig. S8a). From the fitting result (Supplementary Fig. S8b), the calculated μ_{eff} for R-CoOOH is 0.09 μ_{B}, which is close to the theoretical predictions from DFT simulations and reported values for low-spin state Co^{3+} in the literatures[9,10].

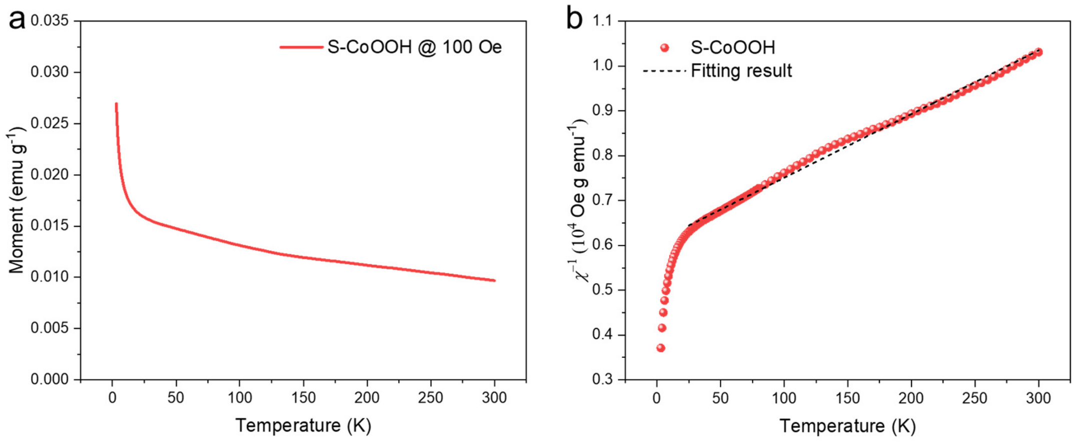

Supplementary Fig. S9 Magnetic property of S-CoOOH. a Temperature-dependent magnetization (**M**-T) under **H** = 100 Oe. **b** The temperature dependence inverse susceptibility (χ^{-1}). The dotted line is the fitting result by a Curie-Weiss law.

The temperature-dependent magnetizations were measured with a magnetic field of **H** = 100 Oe under field-cooling procedures for S-CoOOH (Supplementary Fig. S9a). From the fitting result (Supplementary Fig. S9b), the calculated μ_{eff} for S-CoOOH is 0.76 μ_{B}.

Next, the reasons for the low magnetic moment in S-CoOOH are analyzed. Here, we assume that all Co^{3+} ions in CoOOH are in high-spin states. In this case, the number of unpaired electrons *n* would be 4. The intrinsic magnetic moment could be calculated using the equation: $\mu = \sqrt{n(n+2)}$ where μ represents the intrinsic magnetic moment, and *n* denotes the number of unpaired electrons. And the calculated μ is 4.90 μ_B. At the same time, when all Co^{3+} in CoOOH are in low-spin states without unpaired electrons, the intrinsic magnetic moment is 0. Following this, the proportion of high-spin state Co^{3+} in our S-CoOOH sample is approximately 15 %. This indicates that the low magnetic moment observed in S-CoOOH is due to the low concentration of high-spin state Co^{3+}.

To identify the low concentration of high-spin state Co^{3+} in our S-CoOOH sample, DFT simulations and Co *K*-edge FT-EXAFS were employed. When discussing the DFT calculation results, we have shown that the breakage of Co-O bonds would lead to the formation of coordinatively unsaturated Co atoms (four-coordinated), exhibiting ferromagnetism properties aligned with high-spin state Co^{3+}. Then, the coordination number (CN) of Co-O bond is fitted

based on the Co *K*-edge FT-EXAFS for both S-CoOOH and R-CoOOH. As shown in Supplementary Table S2, it is revealed that the CN of Co-O for S-CoOOH is ~5.6. Hence, the ratio of 4-coordinated Co atoms in S-CoOOH is ~20 %, which agrees well with the proportion of high-spin state Co^{3+} (15%) deduced via magnetic analysis. These results clearly indicate that the low magnetic moment observed in S-CoOOH is due to the low concentration of high-spin state Co^{3+}.

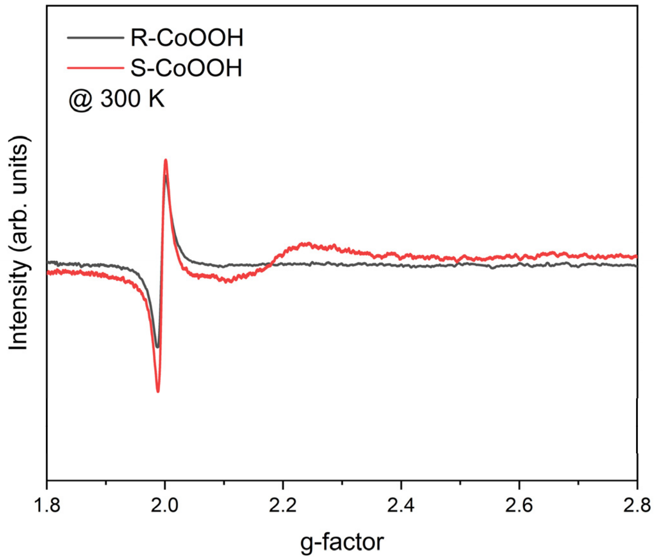

Supplementary Fig. S10 Calculated g-factor obtained at 300 K, extracted from **Fig. 2d.**

Here, we use electron paramagnetic resonance (EPR) technique to study unpaired electrons. g-factor is an important EPR spectroscopy parameter, which tells information about the magnetic nature. It could be calculated from the original data (Fig. 2d), using the following equation: $g = h\nu/\beta\boldsymbol{H}$, where h is the Plank constant with a value of 4.135×10^{-15} eV s, ν is the microwave frequency of X-band spectrometer, β is the electron Bohr magneton with a value of 5.788×10^{-5} eV T^{-1}, and $\boldsymbol{H}$ is the applied magnetic field[11]. Supplementary Fig. S10 is the calculation of g-factor, using Mn as calibration. The peak where g ~ 2.15 only appears in S-CoOOH sample, which corresponds to unpaired electrons in high-spin state Co^{3+} ions (S = 2), as reported in the previous work[12]. Notably, the peak at g ~ 2.00 is shown in both R-CoOOH and S-CoOOH samples. That can be assigned to oxygen vacancies[13].

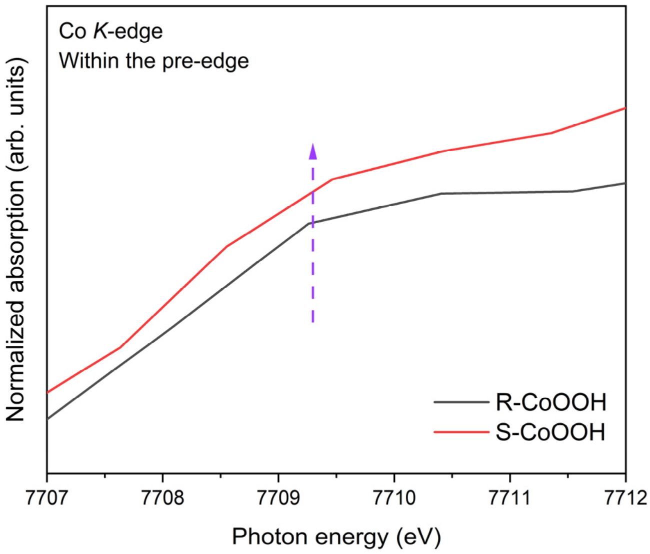

Supplementary Fig. S11 Enlarged Co *K*-edge pre-edge region of R-CoOOH and S-CoOOH, extracted from Fig. 3b.

The high-spin state Co^{3+} in S-CoOOH sample could be further verified by analyzing the electronic configuration of Co^{3+}. As compared with the low-spin state Co^{3+}, the high-spin state Co^{3+} exhibits splitting of 3*d* and 4*p* orbitals[14]. Paired electrons that originally occupy t_{2g}^{*} orbitals in the low-spin state Co^{3+} ($t_{2g}^{6}e_{g}^{0}$) will undergo a transition to different orbitals as a result of an increase in electron pairing energy, ultimately leading to the attainment of the high-spin state Co^{3+} ($t_{2g}^{4}e_{g}^{2}$). Next, S-CoOOH sample is analyzed using Co *K*-edge XAS spectra, with the conventional CoOOH sample (R-CoOOH) as the benchmark (Fig. 3b). For S-CoOOH sample, its white line (7727 to 7732 eV), which is related to the electrons jump from 1*s* to 4*p* orbital, becomes broader with lower intensity, as compared with that of R-CoOOH (as shown in the inset of Fig. 3b). In R-CoOOH, Co^{3+} cations in octahedral sites exhibit a low-spin configuration (LS, $t_{2g}^{6}e_{g}^{0}$), of which 4*p* orbitals are degenerate. At the same time, Co^{3+} follows a high-spin state (HS, $t_{2g}^{4}e_{g}^{2}$), whose 4*p* orbitals become splitting. The variation in the white line indicates that the 4*p* orbitals in S-CoOOH sample experience splitting, providing evidence for the existence of high-spin state Co^{3+}. In addition, when a high-spin state Co^{3+} configuration appears, e_{g}^{*} band would be occupied, indicating an increase of π bonds. This would reduce bond covalency, leading to a decrease in Co valence.

In Co *K*-edge XAS spectra, the pre-edge peak around 7710 eV corresponds to the Co 1*s*-

3*d* transition, and its intensity is correlated to the centrosymmetry of the octahedron in CoOOH. A higher pre-edge peak represents a lower degree of centrosymmetry. For the high-spin state Co^{3+}, the 3*d* band is broader than the low-spin state Co^{3+}, indicating a lower degree of centrosymmetry. Supplementary Fig. S11 shows that the pre-edge peak intensity of S-CoOOH is higher than that of R-CoOOH, which is consistent with our proposal. As a result, in S-CoOOH sample, the high-spin state Co^{3+} appears.

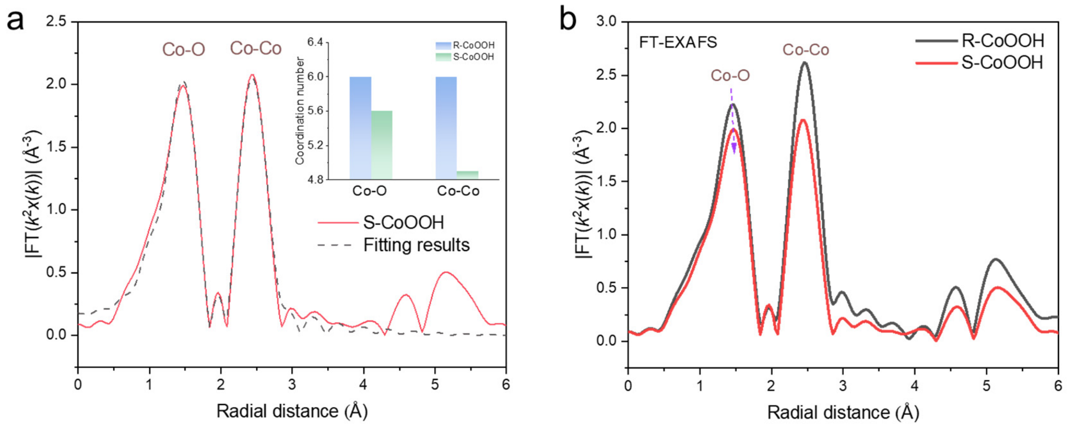

Supplementary Fig. S12 a Fitting result for S-CoOOH (the inset is the corresponding coordination number). **b** Normalized Co *K*-edge Fourier transformed extended X-ray near fine structure (FT-EXAFS) comparison between R-CoOOH and S-CoOOH.

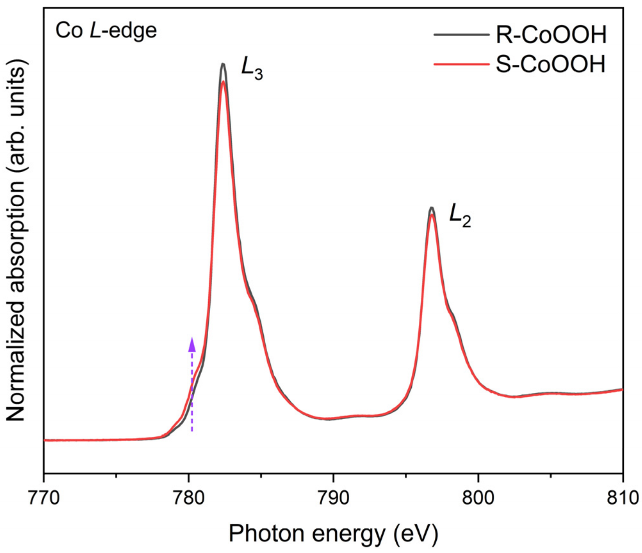

Supplementary Fig. S13 Normalized Co *L*-edge XAS spectra of R-CoOOH and S-CoOOH.

O *K*-edge XAS could provide information about the occupied and unoccupied density of states around Co Fermi level. Specifically, the energy range of 531 to 533 eV reflects electrons jumping from O 1*s* to O 2*p*-Co 3*d* hybrid orbitals[15]. As depicted in Fig. 3c, S-CoOOH sample shows a lower intensity around 531 to 533 eV, which indicates more unoccupied states in Co 3*d* orbitals. Moreover, this peak of S-CoOOH is split into two sub-peaks at 531.5 eV and 532.2 eV (as shown in the inset of Fig. 3c), which are attributed to O 2*p* hybridized with Co 3*d* t_{2g}^* and e_g^* orbitals, respectively. In the comparison between Co^{3+} in low-spin ($t_{2g}^6 e_g^0$) and high-spin ($t_{2g}^4 e_g^2$) states, it is observed that 3*d* electrons that are originally in low-energy t_{2g}^* orbitals partially transfer into high-energy e_g^* orbitals and leave unoccupied states. Hence, the broadening and splitting of peaks around 531 and 533 eV in S-CoOOH, indicate that S-CoOOH is in a high-spin state.

Meanwhile, Co *L*-edge XAS measurements were carried out to study the spin state transition. As depicted in Supplementary Fig. S13, the peak corresponding to high spin at around 780 eV of S-CoOOH is higher than that of R-CoOOH, indicating the emergence of high-spin state Co^{3+}[15]. Moreover, both L_3 and L_2 peaks of S-CoOOH become broader with lower intensity than those of R-CoOOH, which suggests that the 3*d* band becomes broader. This could also be ascribed to the emergence of high-spin state Co^{3+}[16].

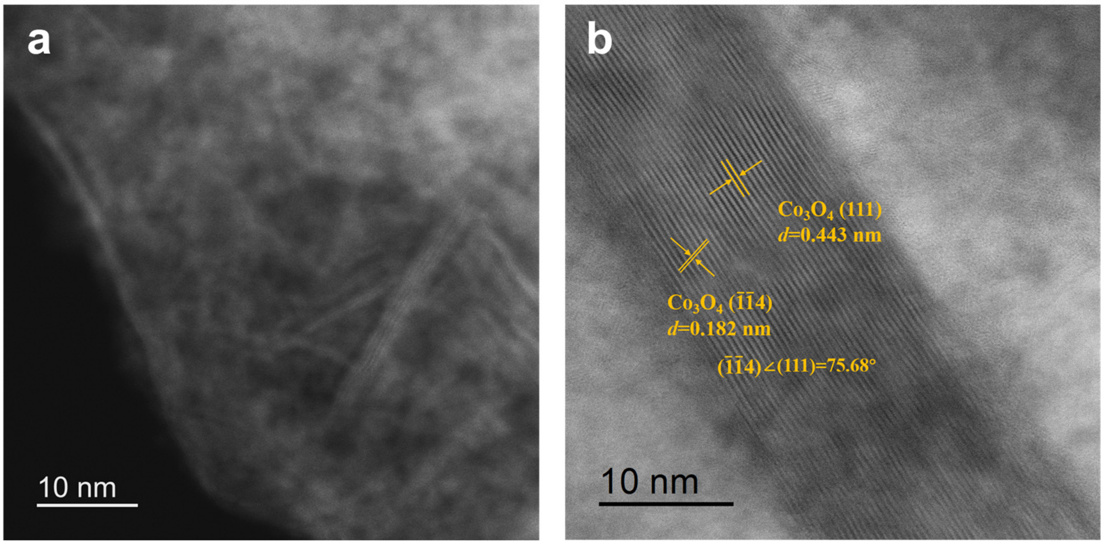

Supplementary Fig. S14 Atomically-resolved STEM images of S-CoOOH. a Atomically-resolved STEM-HAADF (scanning transmission electron microscopy - high-angle annular dark field) image of S-CoOOH. **b** Atomically-resolved STEM-ABF (scanning transmission electron microscopy - annular bright field) image of S-CoOOH.

Supplementary Fig. S14 presents the scanning transmission electron microscopy (STEM) images of S-CoOOH. Supplementary Fig. S14a shows a density of needle-like nanostructures. This is similar to the previously reported nanoribbon (NR) structure $Ni(OH)_2$[17]. It further confirms the rationality of our optimized S-CoOOH model. However, it should be noted that CoOOH is highly electron beam sensitive, and under an electron beam it would transform into Co_3O_4 as shown in Supplementary Fig. S14b. The interlayer spacings are about 0.443 nm and 0.182 nm, well matching the (111) and ($\bar{1}\bar{1}4$) planes of Co_3O_4.

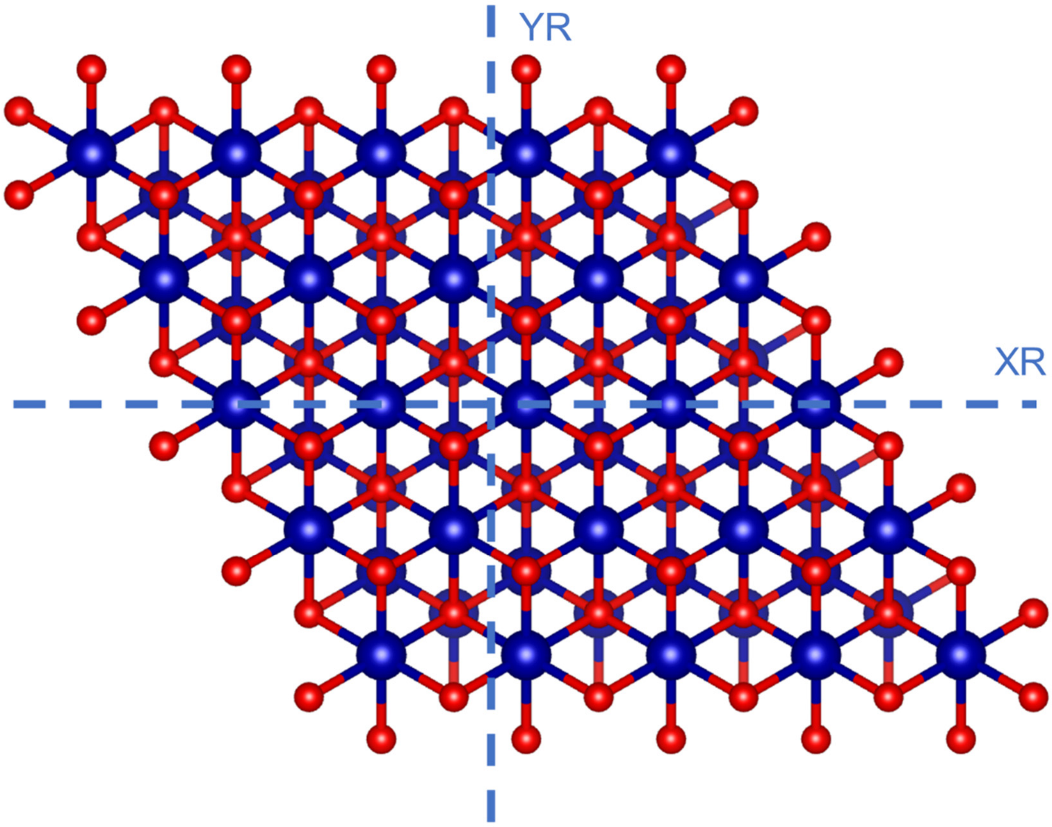

Supplementary Fig. S15 The structure of CoOOH in x-direction (XR) and y-direction (YR).

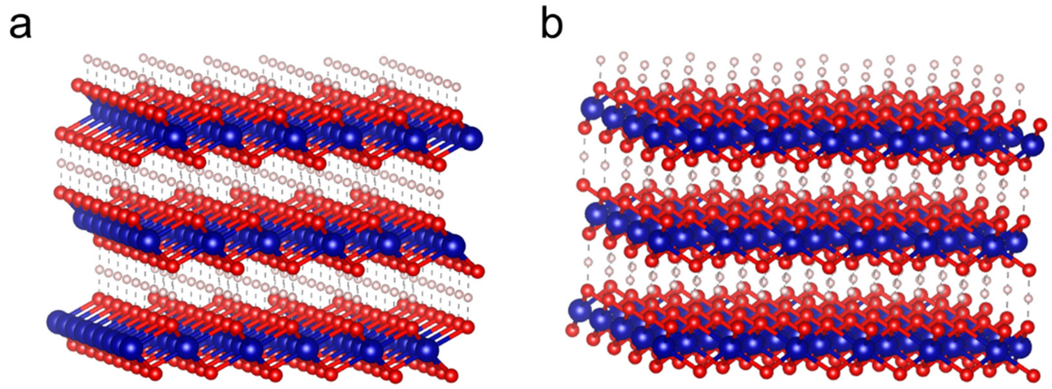

Supplementary Fig. S16 Two different CoOOH edge structures. a CoOOH structure with 3 or 5 coordination at edge. **b** CoOOH structure with 4 coordination at edge.

The CoOOH edge structure can be constructed by cutting along the x-direction (XR) or along the y-direction (YR) of β-CoOOH super cell (Supplementary Fig. S15), which will lead to two different edge structures of CoOOH (Supplementary Fig. S16a. 3 or 5 coordination at edge and Supplementary Fig. S16b. 4 coordination at edge). We compare the energies of these two edge structures by DFT calculations and the results show that the energy of structure with 4 coordination at edge is lower.

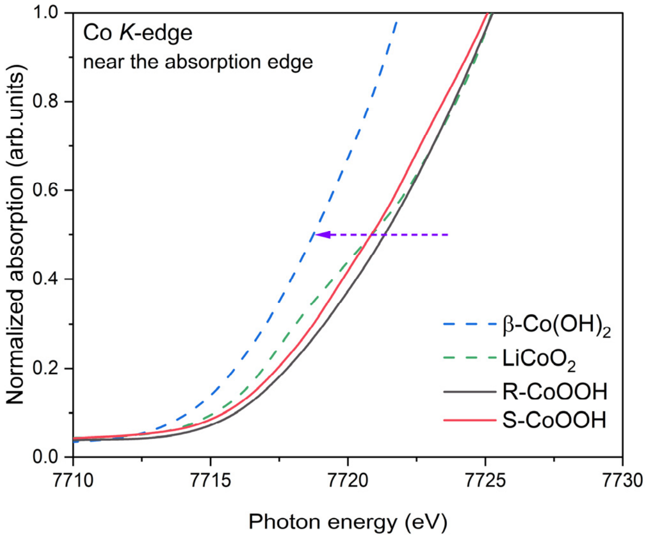

Supplementary Fig. S17 The enlarged results of Co *K*-edge XAS spectra near the adsorption edge.

X-ray absorption spectroscopy (XAS) technique, which provides a powerful bulk-sensitive tool, could be used to evaluate the entire Co electronic and valence state on average. Here, we choose cobalt hydroxide (β-$Co(OH)_2$) and lithium cobalt oxide ($LiCoO_2$) as the benchmark of Co (II) and Co (III). Observed the absorption edge (Supplementary Fig. S17), the oxidation states of both S-CoOOH and R-CoOOH are tracked, showing similar valence of around 3. It excludes the contribution of Co valence states change to spin state rise. Using the commercial $LiCoO_2$ as the reference, both S-CoOOH and R-CoOOH display a similar profile in Co *K*-edge XAS results, indicating that all Co atoms in these CoOOHs are arranged as the edge-sharing CoO_6 octahedral geometry in each layer.

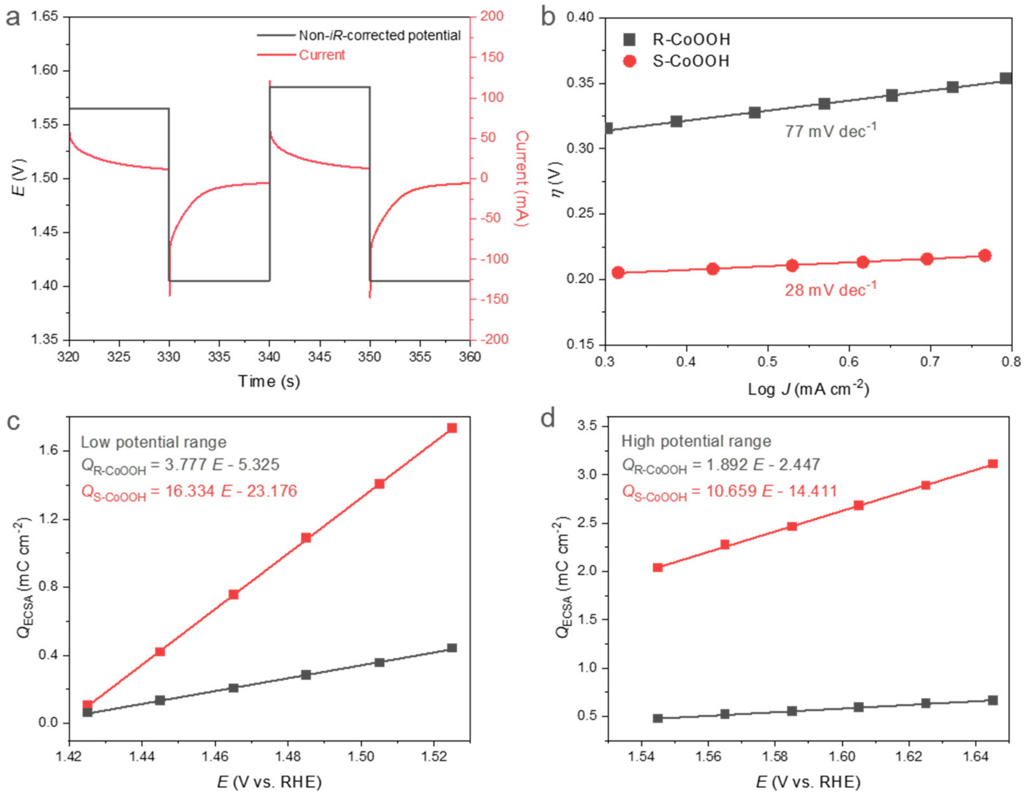

Supplementary Fig. S18 Electron transfer ability comparison between R-CoOOH and S-CoOOH. a Section of the pulse voltammetry protocol (black) showing an oxidative and reductive pulse with the current response (red). **b** Tafel slope derived from OER polarization curves. **c-d** Charge versus log(current) in milliamperes from pulse voltammetry at low potential and at high potential, respectively.

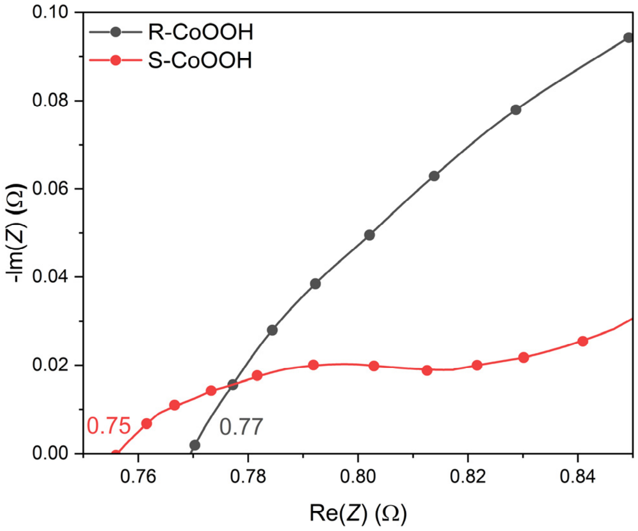

Supplementary Fig. S19 Electrochemical impedance spectroscopy data of R-CoOOH and S-CoOOH.

To exclude the effect of electrolyte resistance on OER performance, 90 % *iR*-corrected results are given in Fig. 5d. The solution resistance R_u exhibits a similar value for both S-CoOOH and R-CoOOH, with 0.75 Ω and 0.77 Ω respectively (Supplementary Fig. S19). OER polarization curves using 90 % *iR*-correction (Fig. 5d) show that the overpotential of S-CoOOH sample at 10 mA cm^{-2} is 226 mV, which is one of the best OER performances reported so far. It displays a 148 mV decrease than that of R-CoOOH.

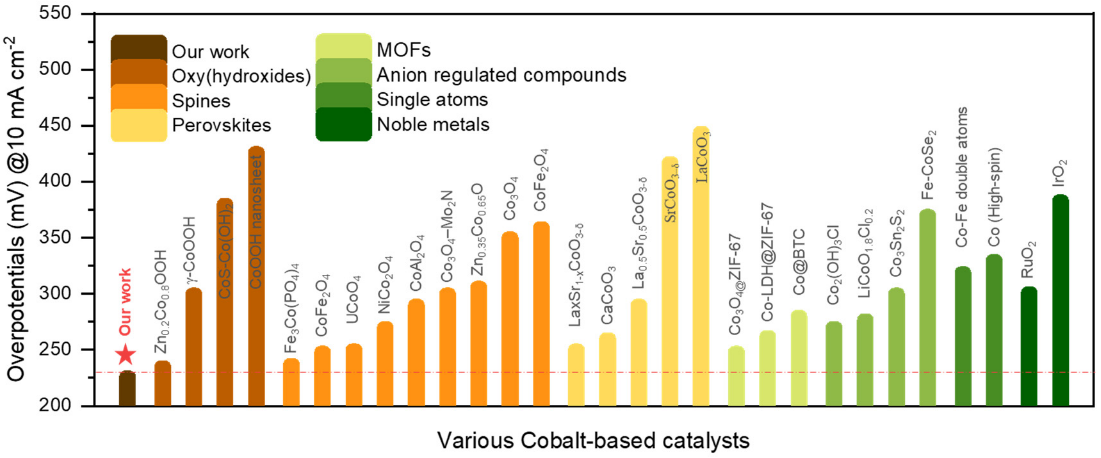

Supplementary Fig. S20 Overpotentials of S-CoOOH benchmarked against reported cobalt-based catalysts (*ref. 21-49*).

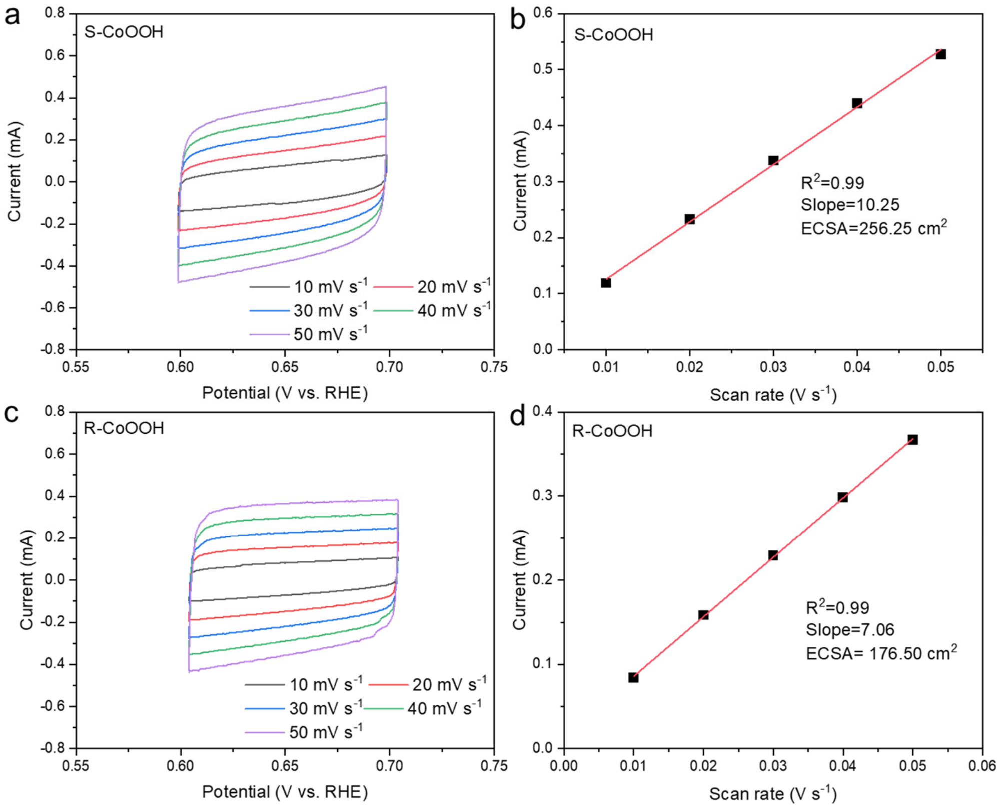

Supplementary Fig. S21 The electrochemical surface area (ECSA) of S-CoOOH and R-CoOOH. a, c CV curves of S-CoOOH and R-CoOOH in 1 M KOH at different scan rates from 10 to 50 mV s^{-1}, charging current densities at 0.65 V versus RHE plotted against the scan rate. **b, d** The current density improvement (Δj) versus scan rate plot for the estimation of ECSA.

Here, we estimate the electrochemical surface area (ECSA) by measuring the non-Faradaic capacitive current associated with double-layer charging from the scan-rate dependence of cyclic voltammograms (CVs)[18]. It can be determined from the double-layer capacitance (C_{DL}) and the specific capacitance (C_{S}) according to the equation: $ECSA = \frac{C_{\mathrm{DL}}}{C_{\mathrm{S}}}$ [19]. The value of C_{S} is typically unknown, and has been a practice to use a single C_{S} value to obtain ECSA for a specific catalyst. Here, C_{S} is evaluated as 0.040 mF cm^{-2} referring to some typical previous reports[19]. Moreover, C_{DL} is the double-layer capacitance and could be extracted from the slope of i_{c} vs. v, by recording CVs at various scan rates within a potential region where no redox processes take place. The resulting ECSA for both S-CoOOH and R-CoOOH are 256.25 cm^2 and 176.50 cm^2, respectively.

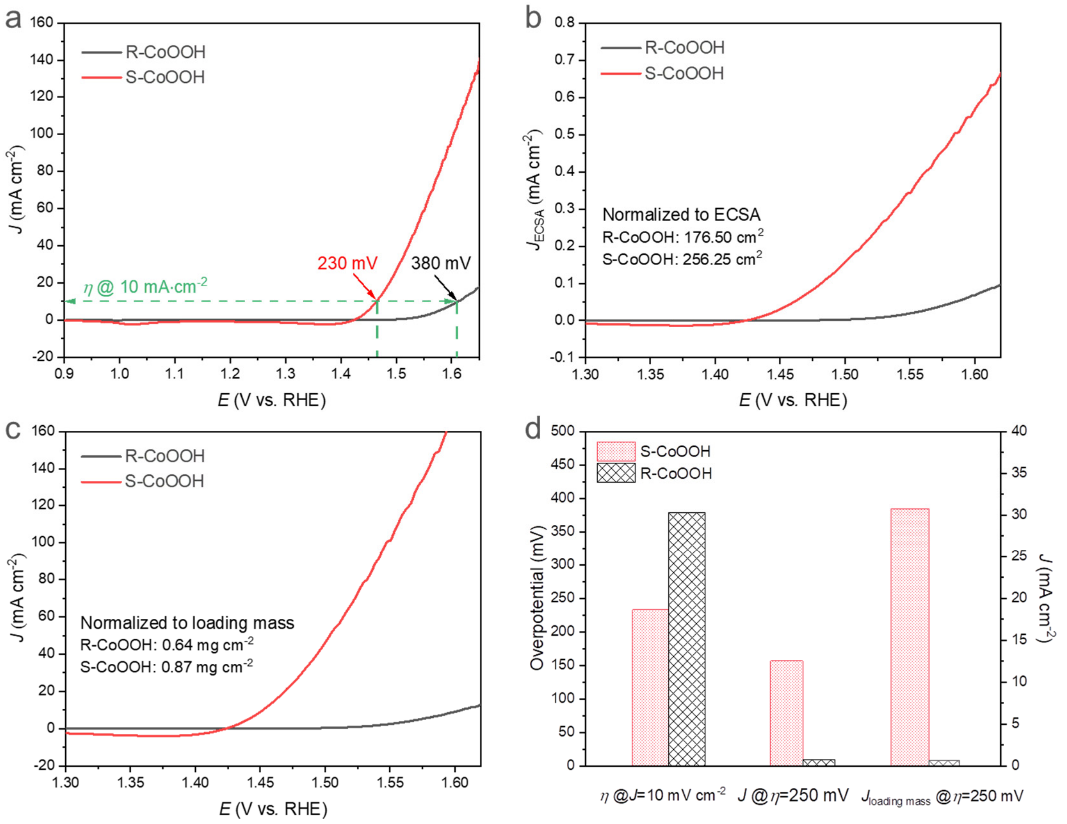

Supplementary Fig. S22 Electrochemical performance of R-CoOOH and S-CoOOH without *iR* correction. a OER curves of CoOOH based on a backward scan conducted at a scan rate of 0.1 mV s^{-1}, overpotentials (η) at 10 mA cm^{-2} for R-CoOOH: 380 mV and S-CoOOH: 230 mV. **b** OER curves normalized to ECSA. **c** OER curves normalized to loading mass. **d** Summary of overpotentials (η) at 10 mA cm^{-2}, current density (J) at 250 mV overpotential, current density normalized to loading mass ($J_{loading\ mass}$) at 250 mV overpotential.

Supplementary Fig. S22a is the raw electrochemical data without *iR* correction of both S-CoOOH and R-CoOOH, based on a backward scan conducted at a scan rate of 0.1 mV s^{-1}. It shows that a potential of 1.462 V (overpotential 230 mV) is required for S-CoOOH sample to reach a current density of 10 mA cm^{-2}, with a 150 mV reduction compared to that of R-CoOOH counterpart. The OER polarization curves normalized to ECSA and loading mass are shown in Supplementary Fig. S22b, c. The specific comparison of catalytic activity between S-CoOOH and R-CoOOH is summarized in Supplementary Fig. S22d.

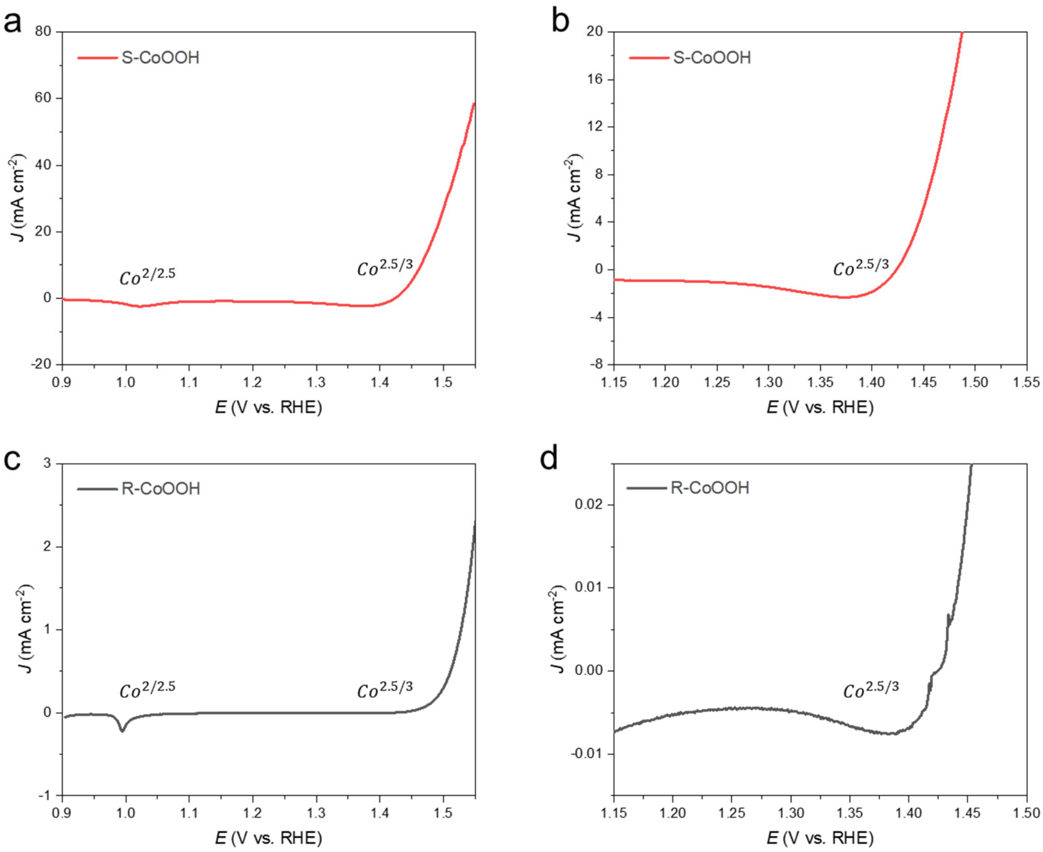

Supplementary Fig. S23 OER curves without *iR* correction, including detailed enlargements of recorded redox peaks. a, b Enlarged view of the OER curves for S-CoOOH. **c, d** Enlarged view of the OER curves for R-CoOOH.

Supplementary Fig. S23 presents a magnified view of the OER curves for S-CoOOH and R-CoOOH, based on a backward scan conducted at a scan rate of 0.1 mV s^{-1}, derived from Supplementary Fig. S22a. In both samples, two distinct redox peaks are evident. For the redox reactions of CoOOH, we find the peak around 1.0 V should be ascribed to the $Co^{2+/2.5+}$ redox process, while the peak around 1.4 V belongs to the $Co^{2.5+/3+}$ redox process. These findings were proposed by William C. Chueh et al.[20], where they used a suite of correlative operando scanning probe and X-ray microscopy techniques to monitor the transition of local operational chemical, physical and electronic nanoscale structure of single-crystalline β-$Co(OH)_2$ platelet particles under anodic potentials. At pre-catalytic voltages, the pre-catalysts transform into α-$CoO_2H_{1.5}\cdot 0.5H_2O$, a result of hydroxide intercalation where the oxidation state of cobalt is +2.5. With an increase in voltage to facilitate oxygen evolution, interlayer water and protons are de-intercalated, resulting in the formation of contracted β-CoOOH particles enriched with Co^{3+}

species. Furthermore, in this Nature publication, the researcher corroborated that Co^{3+} (CoOOH) is the active site for the OER, using operando scanning transmission X-ray microscopy (STXM).

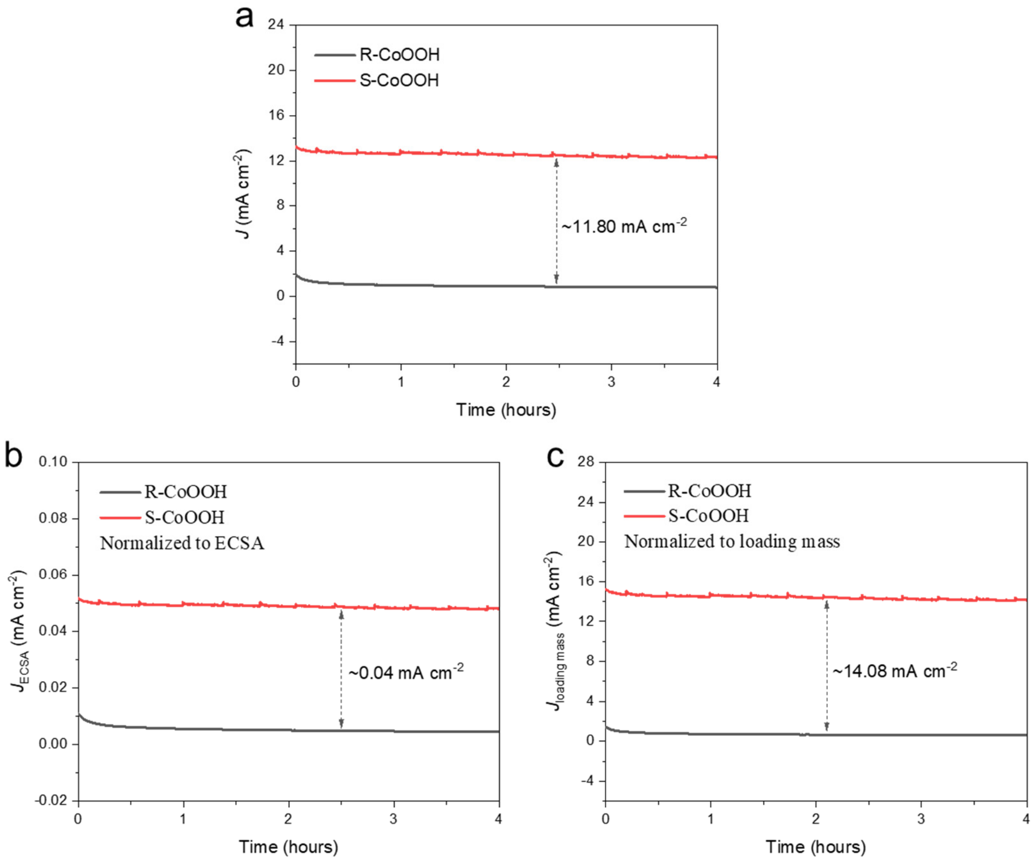

Supplementary Fig. S24 Intrinsic current responses at 1.48 V_{RHE} of R-CoOOH and S-CoOOH. a Chronoamperometry (CA) measurements at the potential of 1.48 vs. reversible hydrogen electrode (RHE) of R-CoOOH and S-CoOOH. **b** Chronoamperometry curves normalized to ECSA. **c** Chronoamperometry curves normalized to loading mass.

Supplementary Fig. S24a-c display the current responses at the fixed potential of 1.48 vs. reversible hydrogen electrode (RHE). They represent the raw data, normalized to electrochemical surface area (ECSA), and loading mass, respectively.

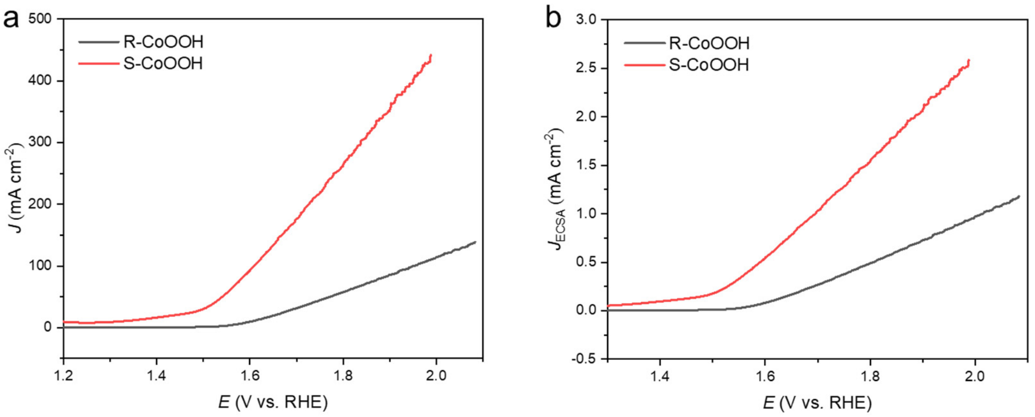

Supplementary Fig. S25 OER activities at high current density for R-CoOOH and S-CoOOH. a OER polarization curves. **b** OER polarization curves normalized to ECSA.

Supplementary Fig. S25 is the electrochemical data of R-CoOOH and S-CoOOH at high current density. To obtain stable high voltage values, we employed a forward sweep during the linear sweep voltammetry (LSV) testing of both R-CoOOH and S-CoOOH. The resulting LSV curves (Supplementary Fig. S25a) clearly showcase that S-CoOOH continues to outperform R-CoOOH at high current density. Moreover, when normalized to the electrochemical surface area (ECSA), the performance of S-CoOOH remains superior (Supplementary Fig. S25b). This affirms that the high-spin state continues to be a dominant factor, even at high-current density.

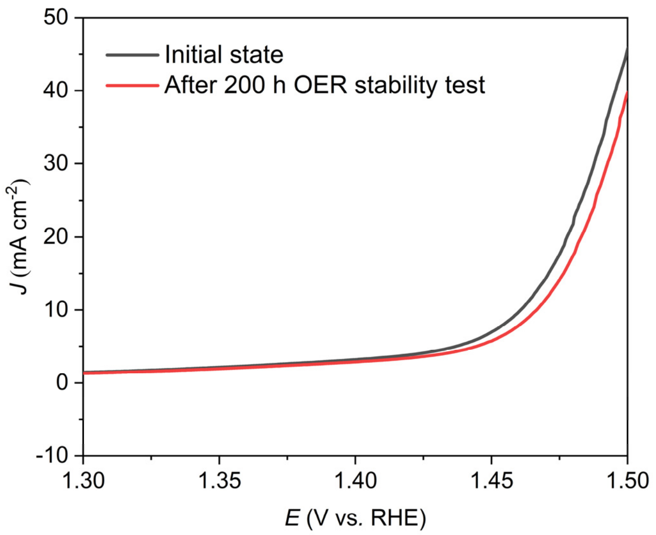

Supplementary Fig. S26 OER polarization curves of S-CoOOH at the initial stage and after a 200 h OER stability test.

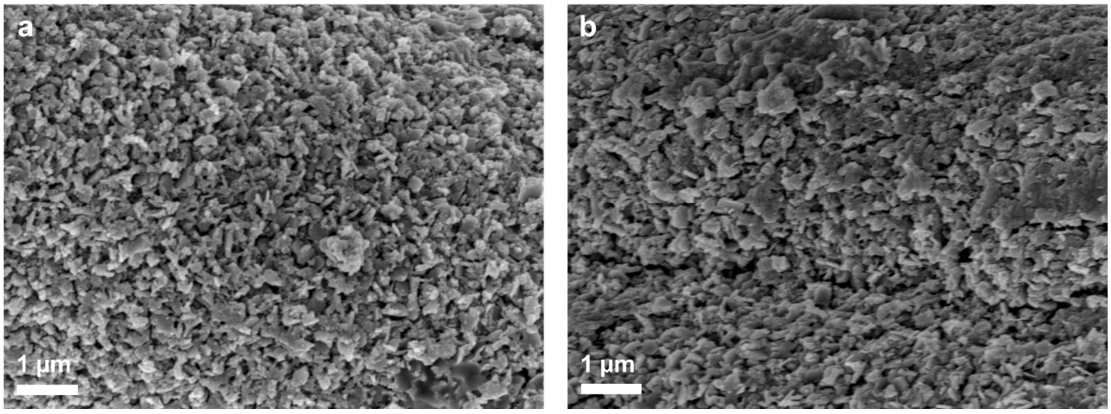

Supplementary Fig. S27 SEM images of S-CoOOH, taken before (**a**) and after (**b**) a 200-hour stability test.

Supplementary Fig. S27 is the SEM images of S-CoOOH taken both before and after a 200-hour stability test. The left image presents the sample in its initial state, while the right one displays its condition following 200 hours of OER testing. A meticulous inspection of the SEM images reveals that S-CoOOH retains its original structural integrity, showcasing no evident changes in morphology.

Supplementary Tables 1-3

Supplementary Table S1. ICP results of S-CoOOH, showing the corresponding mass/atomic ratios

Element	Co	S
Mass ratio (w/w%)	36.4	0.28
Atomic ratio (at%)	98.6	1.40

Supplementary Table S2. FT-EXAFS fitting results of R-CoOOH and S-CoOOH, where CN is the coordination number, σ^2 is the Debye-Waller factor, ΔE_0 is the shift in absorption edge energy, R is the bond length, and R-factor means Residual factor.

Sample	path	CN	σ^2	ΔE_0	R	R-factor
R-CoOOH	Co-O	6	0.0028±0.0012	1.19 (1.29)	1.903±0.010	0.006
	Co-Co	6	0.0044±0.0009	-0.47 (1.27)	2.848±0.008	
S-CoOOH	Co-O	5.6±0.6	0.0026±0.0012	1.96 (1.98)	1.910±0.010	0.007
	Co-Co	4.9±0.6	0.0049±0.0010	-2.05 (2.1)	2.839±0.009	
$Co(OH)_2$ (II)	Co-O	6.1±0.6	0.0060±0.0009	1.69 (1.26)	2.093±0.007	0.007
	Co-Co	6.3±0.7	0.0080±0.0008	3.52 (1.10)	3.188±0.007	
$LiCoO_2$ (III)	Co-O	4.8±0.3	0.0024±0.0005	3.01 (0.90)	1.918±0.004	0.001
	Co-Co	5.7±0.2	0.0028±0.0003	1.21 (0.46)	2.817±0.002	

Supplementary Table S3. OER catalytic performance comparison among S-CoOOH and previously reported cobalt-based catalysts. η stands for overpotential.

Catalyst	Electrolyte	η (mV) at 10 mA cm^{-2}	Tafel slope ($mV\ dec^{-1}$)	Substrate	Year	Ref
Co_3O_4	1 M KOH	350	60.5	Glassy carbon	2023	21
$La_xSr_{1-x}CoO_{3-\delta}$	1 M KOH	250	NA	Glassy carbon	2023	22
CoOOH nanosheet	1 M KOH	426	60	Carbon-coated chip	2022	23
$UCoO_4$	1 M KOH	250	47	Glassy carbon	2022	24
Co_3O_4/Mo_2N	1 M KOH	300	162	Co PBA nanoframes	2022	25
Co_2FeO_4	1 M KOH	359	43 ± 1	Glassy carbon	2022	26
IrO_2	1M KOH	383	82	Carbon cloth	2022	27
$Zn_{0.2}Co_{0.8}OOH$	1 M KOH	235	35.7	Glassy carbon	2021	28
$LiCoO_{1.8}Cl_{0.2}$	1 M KOH	276.8	55.4	Glassy carbon	2021	29
Co-Fe double-atom	1 M KOH	319	40	Carbon cloth	2021	30
Co (High-spin)	1 M KOH	330	70	SiO_2/Si	2021	31
Co_3O_4@ZIF-67	1 M KOH	262	43	ZIF-67 nanoplate	2020	32
$NiCo_2O_4$	1 M KOH	270 ± 3	39	Carbon paper	2020	33
$SrCoO_{3-\delta}$	1 M KOH	417	66	Glassy carbon	2020	34
Fe-$CoSe_2$	1 M KOH	370	53.5	Glassy carbon	2020	35
$Fe_3Co(PO_4)_4$	1 M KOH	237	57	Glassy carbon	2019	36
$CoAl_2O_4$	1 M KOH	290	70	Glassy carbon	2019	37
$MoSe_2$ doped $La_{0.5}Sr_{0.5}CoO_{3-\delta}$	1 M KOH	290	77	Ni mesh	2019	38
$Zn_{0.35}Co_{0.65}O$	1 M KOH	306	42.6	Glassy carbon	2019	39
$CoFe_2O_4$	1 M KOH	248	54.2	Nickel foam	2019	40
$CaCoO_3$	1 M KOH	260	38	Glassy carbon	2019	41
Co@BTC	1 M KOH	280	51	Glassy carbon	2019	42

Co-LDH @ZID-67	1 M KOH	248	52	Carbon cloth	2019	43
$Co_2(OH)_3Cl$	1 M KOH	270	42	Glassy carbon	2019	44
$Co_3Sn_2S_2$	1 M KOH	300	74	Cure wire	2019	45
$La_{0.2}Sr_{0.8}Co_{1-x}Fe_xO_{3-\delta}$	1 M KOH	444	60	Carbon cloth	2019	46
RuO_2	1 M KOH	301	77	Carbon cloth	2019	47
γ-CoOOH	1 M KOH	300	38	Carbon cloth	2017	48
$CoS\text{-}Co(OH)_2$	1 M KOH	380	68	Nickle foam	2016	49

Appendix: All models: Optimized POSCARs (CONTCARs)

R-CoOOH CONTCAR

1.0

4.6295399666	0.0000000000	0.0000000000
3.7450384110	2.7216406086	0.0000000000
3.7450384110	1.2170939432	2.4343397330

Co H O

1 1 2

Cartesian

0.000000000	0.000000000	0.000000000
6.059808394	1.969367276	1.217169867
4.937865045	1.604748731	0.991816927
7.181751021	2.333985586	1.442522661

S-CoOOH CONTCAR

1.0

12.9973001480	0.0000000000	0.0000000000
0.0000000000	4.9534997940	0.0000000000
0.0000000000	0.0000000000	35.7294998169

Co H O

36 36 72

Cartesian

1.624662519	0.000000000	4.276006794
1.624662519	0.000000000	10.006944408
1.624662519	0.000000000	15.690229902
1.624662519	2.476749897	0.039559579
1.624662519	2.476749897	5.718141380
1.624662519	2.476749897	11.450441700
5.958538908	1.648130132	4.278982173
5.957009651	1.646351241	10.008692846
5.953626151	1.646118583	15.686403997
5.957196354	4.126660101	0.042993152
5.958430837	4.123862592	5.721597793
5.958042326	4.122832755	11.451058232
10.289428831	0.826839545	0.042993152
10.288194735	0.829637128	5.721597793
10.288583634	0.830666818	11.451058232
10.288086277	3.305369662	4.278982173
10.289615534	3.307148553	10.008692846
10.292999421	3.307381211	15.686403997
1.624662519	0.000000000	1.377513063
1.624662519	0.000000000	7.144717585
1.624662519	0.000000000	12.857850042

1.624662519	2.476749897	2.869733818
1.624662519	2.476749897	8.580007229
1.624662519	2.476749897	14.352909214
5.957056133	1.650197189	1.379672523
5.957885449	1.646672770	7.146652899
5.957845165	1.646188705	12.857644532
5.957722375	4.125889790	2.873018528
5.957308685	4.123698137	8.581216334
5.955865033	4.123435954	14.351859299
10.288902810	0.827609857	2.873018528
10.289317274	0.829801657	8.581216334
10.290760540	0.830063693	14.351859299
10.289569052	3.303302605	1.379672523
10.288740123	3.306827024	7.146652899
10.288780408	3.307311236	12.857644532
8.123312593	0.000000000	4.293352213
8.123312593	0.000000000	10.011862821
8.123312593	0.000000000	15.795677103
8.123312593	2.476749897	35.663240199
8.123312593	2.476749897	5.714199409
8.123312593	2.476749897	11.438333608
12.455414146	1.653066886	4.290957429
12.454959397	1.653760432	10.015030667
12.452187522	1.654617400	15.796838824
12.453251959	4.127699091	35.665846883
12.455961858	4.130631826	5.711237606
12.456501824	4.131156192	11.438111060
3.793373226	0.825800629	35.665846883
3.790662940	0.822867895	5.711237606
3.790123361	0.822343306	11.438111060

3.791210652	3.300433055	4.290957429
3.791665401	3.299739509	10.015030667
3.794436888	3.298882689	15.796838824
8.123312593	0.000000000	1.516494306
8.123312593	0.000000000	7.148498768
8.123312593	0.000000000	12.895571364
8.123312593	2.476749897	2.835203236
8.123312593	2.476749897	8.579890098
8.123312593	2.476749897	14.215400221
12.458265040	1.650631208	1.515145309
12.455193356	1.654611052	7.148013209
12.458829021	1.653417055	12.895162473
12.455253783	4.128637696	2.836189527
12.454032082	4.130946564	8.576900610
12.460568996	4.128769969	14.210953524
3.791371402	0.824862024	2.836189527
3.792593103	0.822553083	8.576900610
3.786055802	0.824729751	14.210953524
3.788360145	3.302868586	1.515145309
3.791431054	3.298888889	7.148013209
3.787795777	3.300082887	12.895162473
6.921583299	4.952180019	4.295411579
6.920826417	4.949236361	10.012784957
6.917604442	0.117505236	15.783517899
6.918582113	2.596814844	35.679764108
6.920463083	2.471309148	5.714253715
6.920840362	2.473943235	11.436611791
11.251148486	1.655786449	4.295495700
11.252185034	1.655243924	10.015276640
11.256609342	1.775293518	15.782164511

11.254259679	4.250742537	35.684440806
11.250640283	4.132427546	5.712917896
11.251967343	4.137326360	11.437077118
2.584679771	0.949393021	35.679199752
2.587566108	0.823282133	5.710588597
2.588074504	0.827114056	11.435971833
2.587753778	3.302915826	4.291228426
2.588102974	3.299645324	10.014294875
2.581837980	3.424293166	15.783723410
9.325041886	0.001319683	4.295411579
9.325798768	0.004263457	10.012784957
9.329020743	4.835994678	15.783517899
9.328043072	2.356684950	35.679764108
9.326162102	2.482190793	5.714253715
9.325784823	2.479556559	11.436611791
0.661571259	1.650584116	4.291228426
0.661222160	1.653854618	10.014294875
0.667487057	1.529206775	15.783723410
0.664645218	4.004106773	35.679199752
0.661758929	4.130217588	5.710588597
0.661250581	4.126385517	11.435971833
4.992365119	0.702757036	35.684440806
4.995985289	0.821072101	5.712917896
4.994658229	0.816173212	11.437077118
4.995477087	3.297713493	4.295495700
4.994440539	3.298255870	10.015276640
4.990016230	3.178206571	15.782164511
6.943338409	0.039536375	1.493151543
6.921618935	4.950800013	7.148999767
6.917889532	4.946280302	12.891515457

6.917340270	2.474190066	2.841308926
6.920747398	2.473623330	8.579508359
6.942710128	2.512273683	14.239159596
11.275862173	1.692194661	1.492924869
11.252539071	1.657175460	7.150116233
11.248244137	1.656173229	12.890714711
11.248039617	4.129244143	2.842338342
11.253400537	4.132984390	8.577386169
11.276501300	4.173411420	14.234035672
2.584325540	0.824902104	2.839146538
2.587662945	0.823501505	8.576095605
2.610960288	0.867116218	14.236776524
2.610882237	3.343628068	1.490257223
2.587574629	3.300787062	7.147170935
2.584344907	3.300848769	12.891045870
9.303286776	4.913963539	1.493151543
9.325006250	0.002699924	7.148999767
9.328735653	0.007219590	12.891515457
9.329284915	2.479309728	2.841308926
9.325877787	2.479876316	8.579508359
9.303915057	2.441225963	14.239159596
0.638442751	1.609872021	1.490257223
0.661750456	1.652712880	7.147170935
0.664980081	1.652651025	12.891045870
0.664999497	4.128597542	2.839146538
0.661662189	4.129998216	8.576095605
0.638364797	4.086383355	14.236776524
4.998585568	0.824255503	2.842338342
4.993224648	0.820515330	8.577386169
4.970123885	0.780088227	14.234035672

4.970763012	3.261305428	1.492924869
4.994086114	3.296324334	7.150116233
4.998381435	3.297326713	12.890714711

Supplementary References:

1 Gao, Z. Y. et al. Porous $Co_3S_4@Ni_3S_4$ heterostructure arrays electrode with vertical electrons and ions channels for efficient hybrid supercapacitor. *Chem. Eng. J.* **343**, 572-582 (2018).

2 Liu, Y. W. et al. Ultrathin Co_3S_4 nanosheets that synergistically engineer spin states and exposed polyhedra that promote water oxidation under neutral conditions. *Angew. Chem.* **127**, 11383-11387 (2015).

3 Jing, C. et al. Electrocatalyst with dynamic formation of the dual-active site from the dual pathway observed by *in situ* Raman spectroscopy. *ACS Catal.* **12**, 10276-10284 (2022).

4 Hausmann, J. N. et al. Understanding the formation of bulk- and surface-active layered (oxy)hydroxides for water oxidation starting from a cobalt selenite precursor. *Energy Environ. Sci.* **13**, 3607-3619 (2020).

5 Fan, K. et al. Direct observation of structural evolution of metal chalcogenide in electrocatalytic water oxidation. *ACS Nano* **12**, 12369-12379 (2018).

6 Chen, K. et al. Evidence of the anomalous fluctuating magnetic state by pressure-driven 4f valence change in $EuNiGe_3$. *J. Phys. Chem. Lett.* **14**, 1000-1006 (2023).

7 Du, X.-J. et al. [Ba_4Cl] cations directed perovskite-like polar metal formate frameworks $\{[Ba_4Cl][M_3(HCO_2)_{13}]\}_n$ (M = Mn, Co, and Mg): microwave-assisted synthesis, structures, and properties. *Inorg. Chem.* **61**, 2265-2271 (2022).

8 Lee, N. et al. Highly nonlinear magnetoelectric effect in buckled-honeycomb antiferromagnetic $Co_4Ta_2O_9$. *Sci. Rep.* **10**, 12362 (2020).

9 Zhou, S. M. et al. Engineering electrocatalytic activity in nanosized perovskite cobaltite through surface spin-state transition. *Nat. Commun.* **7**, 11510 (2016).

10 Zhang, Z. R. et al. Regulating spin states in oxygen electrocatalysis. *Angew. Chem. Int. Ed.* **62**, e202216837 (2023).

11 Bonke, S. A., Risse, T., Schnegg, A. & Brückner, A. In situ electron paramagnetic resonance spectroscopy for catalysis. *Nat. Rev. Methods Primers* **1**, 34 (2021).

12 Varghese, M. et al. Room-temperature mixed spin state of Co^{3+} in $Sr_2Co_{0.02}Ga_{0.98}NbO_6$ double perovskites: combined NMR and EPR studies in a potential inorganic pigment.

J. Phys. Chem. C **126**, 8450-8460 (2022).

13 Carvalho, R. P. D. et al. Electron paramagnetic resonance analysis of active bio-Pd-based electrodes for fuel cells. *Adv. Mater. Res.* **71-73**, 737-740 (2009).

14 Sun, Y. M. et al. Engineering high-spin state cobalt cations in spinel zinc cobalt oxide for spin channel propagation and active site enhancement in water oxidation. *Angew. Chem. Int. Ed.* **60**, 14536-14544 (2021).

15 Wu, C. R. et al. Tuning the spin state of Co^{3+} by crystal facet engineering for enhancing the oxygen evolution reaction activity. *Chem. Mater.* **34**, 10509-10516 (2022).

16 Zhong, H. Y. et al. Optimization of oxygen evolution activity by tuning e_g^* band broadening in nickel oxyhydroxide. *Energy Environ. Sci.* **16**, 641-652 (2023).

17 Wang, X. P. et al. Strain stabilized nickel hydroxide nanoribbons for efficient water splitting. *Energy Environ. Sci.* **13**, 229-237 (2020).

18 Morales, D. M. & Risch, M. Seven steps to reliable cyclic voltammetry measurements for the determination of double layer capacitance. *J. Phys. Energy* **3**, 034013 (2021).

19 McCrory, C. C., Jung, S., Peters, J. C. & Jaramillo, T. F. Benchmarking heterogeneous electrocatalysts for the oxygen evolution reaction. *J. Am. Chem. Soc.* **135**, 16977-16987 (2013).

20 Mefford, J. T. et al. Correlative operando microscopy of oxygen evolution electrocatalysts. *Nature* **593**, 67-73 (2021).

21 Zhang, R. R. et al. Tracking the role of defect types in Co_3O_4 structural evolution and active motifs during oxygen evolution reaction. *J. Am. Chem. Soc.* **145**, 2271-2281 (2023).

22 Lu, M. et al. Artificially steering electrocatalytic oxygen evolution reaction mechanism by regulating oxygen defect contents in perovskites. *Sci. Adv.* **8**, eabq3563 (2022).

23 Wang, S. H. et al. Identifying the geometric catalytic active sites of crystalline cobalt oxyhydroxides for oxygen evolution reaction. *Nat. Commun.* **13**, 6650 (2022).

24 Lin, X. et al. 5f covalency synergistically boosting oxygen evolution of $UCoO_4$ Catalyst. *J. Am. Chem. Soc.* **144**, 416-423 (2022).

25 Wang, T. T. et al. Nanoframes of Co_3O_4–Mo_2N heterointerfaces enable high-performance bifunctionality toward both electrocatalytic HER and OER. *Adv. Funct.*

Mater. **32**, 2107382 (2021).

26 Xiang, W. K. et al. 3D atomic-scale imaging of mixed Co-Fe spinel oxide nanoparticles during oxygen evolution reaction. *Nat. Commun.* **13**, 179 (2022).

27 Shah, K. et al. Cobalt single atom incorporated in ruthenium oxide sphere: a robust bifunctional electrocatalyst for HER and OER. *Angew. Chem. Int. Ed.* **61**, e202114951 (2022).

28 Huang, Z.-F. et al. Chemical and structural origin of lattice oxygen oxidation in Co–Zn oxyhydroxide oxygen evolution electrocatalysts. *Nat. Energy* **4**, 329-338 (2019).

29 Wang, J. et al. Redirecting dynamic surface restructuring of a layered transition metal oxide catalyst for superior water oxidation. *Nat. Catal.* **4**, 212-222 (2021).

30 Bai, L. C. et al. Double-atom catalysts as a molecular platform for heterogeneous oxygen evolution electrocatalysis. *Nat. Energy* **6**, 1054-1066 (2021).

31 Li, Z. J. et al. Tuning the spin density of cobalt single-atom catalysts for efficient oxygen evolution. *ACS Nano* **15**, 7105-7113 (2021).

32 Zhang, S. L. et al. Metal atom-doped Co_3O_4 hierarchical nanoplates for electrocatalytic oxygen evolution. *Adv. Mater.* **32**, 2002235 (2020).

33 Li, J. T. et al. Boosted oxygen evolution reactivity by igniting double exchange interaction in spinel oxides. *J. Am. Chem. Soc.* **142**, 50-54 (2020).

34 Pan, Y. L. et al. Direct evidence of boosted oxygen evolution over perovskite by enhanced lattice oxygen participation. *Nat. Commun.* **11**, 2002 (2020).

35 Dou, Y. H. et al. Approaching the activity limit of $CoSe_2$ for oxygen evolution via Fe doping and Co vacancy. *Nat. Commun.* **11**, 1664 (2020).

36 Sultan, S. et al. Superb water splitting activity of the electrocatalyst $Fe_3Co(PO_4)_4$ designed with computation aid. *Nat. Commun.* **10**, 5195 (2019).

37 Wu, T. Z. et al. Iron-facilitated dynamic active-site generation on spinel $CoAl_2O_4$ with self-termination of surface reconstruction for water oxidation. *Nat. Catal.* **2**, 763-772 (2019).

38 Oh, N. K. et al. In-situ local phase-transitioned $MoSe_2$ in $La_{0.5}Sr_{0.5}CoO_{3\text{-delta}}$ heterostructure and stable overall water electrolysis over 1000 hours. *Nat. Commun.* **10**, 1723 (2019).

39 Wahl, S. et al. $Zn_{0.35}Co_{0.65}O$ – a stable and highly active oxygen evolution catalyst formed by Zinc leaching and tetrahedral coordinated Cobalt in Wurtzite structure. *Adv. Energy Mater.* **9**, 1900328 (2019).

40 Huang, L. L. et al. Zirconium-regulation-induced bifunctionality in 3D cobalt-iron oxide nanosheets for overall water splitting. *Adv. Mater.* **31**, 1901439 (2019).

41 Li, X. et al. Exceptional oxygen evolution reactivities on $CaCoO_3$ and $SrCoO_3$. *Sci. Adv.* **5**, eaav6262 (2019).

42 Tripathy, R. K., Samantara, A. K. & Behera, J. N. A cobalt metal-organic framework (Co-MOF): a bi-functional electro active material for the oxygen evolution and reduction reaction. *Dalton Trans.* **48**, 10557-10564 (2019).

43 Chen, Z. L. et al. Oriented transformation of Co-LDH into 2D/3D ZIF-67 to achieve Co–N–C hybrids for efficient overall water splitting. *Adv. Energy Mater.* **9**, 1803918 (2019).

44 Jiang, H. L. et al. Tracking structural self-reconstruction and identifying true active sites toward cobalt oxychloride precatalyst of oxygen evolution reaction. *Adv. Mater.* **31**, 1805127 (2019).

45 Li, G. W. et al. Surface states in bulk single crystal of topological semimetal $Co_3Sn_2S_2$ toward water oxidation. *Sci. Adv.* **5**, eaaw9867 (2019).

46 Kim, B.-J. et al. Functional role of Fe-doping in Co-based perovskite oxide catalysts for oxygen evolution reaction. *J. Am. Chem. Soc.* **141**, 5231-5240 (2019).

47 Lu, X. F. et al. Interfacing manganese oxide and cobalt in porous graphitic carbon polyhedrons boosts oxygen electrocatalysis for Zn-air batteries. *Adv. Mater.* **31**, 1902339 (2019).

48 Seh, Z. W. et al. Combining theory and experiment in electrocatalysis: Insights into materials design. *Science* **355**, 146 (2017).

49 Yoon, T. & Kim, K. S. One-step synthesis of CoS-doped β-$Co(OH)_2$@amorphous MoS_{2+x} hybrid catalyst grown on nickel foam for high-performance electrochemical overall water splitting. *Adv. Funct. Mater.* **26**, 7386-7393 (2016).
